# 7‐Epitaxol induces apoptosis in cisplatin‐resistant head and neck squamous cell carcinoma via suppression of AKT and MAPK signalling

**DOI:** 10.1111/jcmm.17602

**Published:** 2022-10-29

**Authors:** Hui‐Ju Yang, Bharath Kumar Velmurugan, Mu‐Kuan Chen, Chia‐Chieh Lin, Yu‐Sheng Lo, Yi‐Ching Chuang, Hsin‐Yu Ho, Ming‐Ju Hsieh, Jiunn‐Liang Ko

**Affiliations:** ^1^ Institute of Medicine Chung Shan Medical University Taichung Taiwan; ^2^ Department of Dermatology Changhua Christian Hospital Changhua Taiwan; ^3^ Faculty of Applied Sciences Ton Duc Thang University Ho Chi Minh City Vietnam; ^4^ Department of Otorhinolaryngology, Head and Neck Surgery Changhua Christian Hospital Changhua Taiwan; ^5^ Oral Cancer Research Center Changhua Christian Hospital Changhua Taiwan; ^6^ Ph.D. Program in Tissue Engineering and Regenerative Medicine College of Medicine, National Chung Hsing University Taichung Taiwan; ^7^ Graduate Institute of Biomedical Sciences China Medical University Taichung Taiwan; ^8^ Department of Medical Oncology and Chest Medicine Chung Shan Medical University Hospital Taichung Taiwan

**Keywords:** 7‐Epitaxol, apoptosis, cisplatin, head and neck squamous cell carcinoma

## Abstract

Head and neck squamous cell carcinoma (HNSCC) is the sixth most common cancer worldwide. Although cisplatin‐based chemotherapy is commonly used in HNSCC, frequent development of cisplatin resistance is a potential cause of poor HNSCC prognosis. In the present study, we investigated the anticancer efficacy of a major paclitaxel metabolite namely 7‐Epitaxol in cisplatin‐resistant HNSCC. The findings revealed that 7‐Epitaxol exerts cytotoxic effects in cisplatin‐resistant HNSCC cell lines by inducing cell cycle arrest and intrinsic and extrinsic apoptotic pathways. Specifically, 7‐Epitaxol increased Fas, TNF‐R1, DR5, DcR3 and DcR2 expressions, reduced Bcl‐2 and Bcl‐XL (anti‐apoptotic proteins) expressions, and increased Bid and Bim L/S (pre‐apoptotic proteins) expressions, leading to activation of caspase‐mediated cancer cell apoptosis. At the upstream cell signalling level, 7‐Epitaxol reduced the phosphorylation of AKT, ERK1/2 and p38 to trigger apoptosis. In vivo results showed that animals treated with 7‐Epitaxol show antitumor growth compared to control animals. Taken together, the study demonstrates the potential anticancer efficacy of 7‐Epitaxol in inducing apoptosis of cisplatin‐resistant HNSCC cells through the suppression of AKT and MAPK signalling pathways.

## 1 INTRODUCTION

Head and neck squamous cell carcinoma (HNSCC) refers to a group of cancers that originate primarily from the mucosal epithelium in the oral cavity, pharynx and larynx. These types of cancers can occur due to excessive consumption of tobacco and alcohol, or due to infection with human papilloma virus (HPV). Regarding prevalence, HNSCC is considered to be the 6th most common cancer worldwide, with 890,000 new cases and 450,000 deaths in 2018.^1^ Although the 5‐year survival rate of HPV‐positive cancers has improved considerably over the past three decades,^2^ no such improvement has been observed in older patients and those with HPV‐negative cancers.^3^

Surgery along with chemotherapy or radiation therapy is considered to be the gold‐standard for treating HNSCC patients.^4^ In patients with recurrent or metastatic HNSCC, treatment with monoclonal antibodies targeting epidermal growth factor receptor (EGFR), such as cetuximab, has shown promising outcomes when used in combination with radiation therapy.^5^ However, studies have identified cetuximab as a less potent radiosensitizer than cisplatin in treating HPV‐positive cancer patients.^6^ Cisplatin‐based chemotherapy is considered as the standard of care treatment for patients with HNSCC.^7^ However, in locally advanced HNSCC, cisplatin treatment has been found to associate with limited efficacy and severe acute and late persistent toxicity.^8^ Another disadvantage of cisplatin‐based chemotherapy is frequent development of drug resistance, which is associated with reduced treatment efficacy and poor disease prognosis.^9^, ^10^ For cisplatin‐refractory recurrent or metastatic HNSCC, immune checkpoint inhibitors pembrolizumab and nivolumab have shown promising outcomes in clinical trials.^11^, ^12^, ^13^ Despite advancement in therapeutic interventions, no significant change in overall prognosis has been observed particularly for HPV‐negative HNSCC.^4^ Moreover, because of high treatment expenses, most of the targeted therapies, including immunotherapy, remain unavailable or inaccessible in many countries across the world.^8^

Plant‐derived bioactive compounds, also known as phytochemicals, have opened a new path towards prophylactic and therapeutic management of various cancer types, including HNSCC.^14^ Paclitaxel, also known as Taxol, is a widely used natural chemotherapeutic agent derived from the evergreen tree, *Taxus brevifolia*.^15^ This tricyclic diterpenoid compound has been found to exert anticancer effects in various types of cancer, including breast, ovarian, lung and brain cancers.^16^, ^17^, ^18^, ^19^ 7‐Epitaxol is the major bioactive metabolite of paclitaxel, which is more stable and cytotoxic to cancer cells than paclitaxel.^20^ In this context, one recent study has shown that the treatment with 7‐Epitaxol induces apoptosis and autophagy in HNSCC via ERK signalling pathway.^21^ Another study investigating the efficacy of mesenchymal stem cells (MSCs) for targeted drug delivery has revealed that MSCs incorporated with paclitaxel are capable of producing and releasing 7‐Epitaxol together with paclitaxel without changing their biological activity.^22^ These findings highlight that targeted delivery of 7‐Epitaxol as an anticancer agent is possible via MSCs.

Despite being a superior anticancer agent than paclitaxel, anticancer properties of 7‐Epitaxol have not been studied widely. In the present, we investigated the anticancer effect as well as mode of action of 7‐Epitaxol in cisplatin‐resistant HNSCC.

## 2 MATERIALS AND METHODS

### 2.1 Chemical

7‐Epitaxol (7‐E) (≥98% purity) purchased from ChemFaces (Wuhan, Hubei, China) was dissolved in dimethyl sulfoxide (DMSO) to prepare 100 mM stock solution.^21^ The stock was further diluted to prepare working solutions of 0, 25, 50 and 100 nM concentrations. The final concentration of DMSO in the working solutions was less than 0.2%. Other chemical reagents used in the study including 3‐(4,5‐dimethylthiazol‐2‐yl)‐2,5‐diphenyltetrazolium bromide (MTT), propidium iodide (PI), RNase A, DAPI dye, protease inhibitor cocktail and phosphatase inhibitor cocktail were obtained from Sigma‐Aldrich (St Louis, MO). The primary antibodies were purchased from Cell Signaling Technology (Danvers, MA). Specific inhibitor for AKT (LY294002), ERK1/2 (U0126), p38 (SB203580) and JNK1/2 (SP600125) were purchased from Santa Cruz Biotechnology (Santa Cruz, CA).

### 2.2 Cell culture

Two HNSCC cell lines including SCC‐9 and SAS (Japanese Collection of Research Bioresources Cell Bank, JCRB Cell Bank) were cultured in Dulbecco's Modified Eagle Medium‐F12 (DMEM; Life Technologies, Grand Island, NY) supplemented with 10% foetal bovine serum, 0.1 mM nonessential amino acids, 1 mM glutamine, 1% penicillin/streptomycin (10,000 U/ml penicillin and 10 mg/ml streptomycin), 1.5 g/L sodium bicarbonate and 1 mM sodium pyruvate. The cells were maintained at 37°C in a humidified atmosphere of 5% CO_2._ The cisplatin‐resistant cell line was established by continuous culturing of the cells with increasing concentrations of cisplatin (1–1000 nM) for 6 months. The culture medium of cells was supplemented with 1 μM cisplatin to maintain drug resistance.

### 2.3 Cell cytotoxicity

The cells were cultured in 96‐well plates at a density of 1 × 10^4^ cells/well overnight, followed by incubation with different concentrations of 7‐Epitaxol (0, 25, 50 or 100 nM) for 24, 48 or 72 h. Upon completion of the treatment, 20 ul of MTT (5 mg/ml) solution was added to each well and incubated for 4 h at 37°C. The blue formazan crystals formed were dissolved in DMSO, and the absorbance was measured at 595 nm using spectrophotometry. The entire procedure was repeated three times using the same conditions to obtain three independent experimental replicates.

### 2.4 Colony formation assay

The cell lines were seeded onto 6‐well plates at a density of 5 × 10^3^ cells/well and cultured overnight, followed by incubation with different concentrations of 7‐Epitaxol (0, 25, 50 and 100 nM). The incubation medium was changed every 3 days. After 2 weeks, the colonies were fixed with 4% paraformaldehyde and then stained with 0.3% crystal violet solution. The stained colonies were dissolved in DMSO and counted by a stereomicroscope as previously described.^21^

### 2.5 Cell cycle analysis

The cell lines were seeded onto 6‐well plates at a density of 5 × 10^5^ cells/well and cultured overnight. The cells were next incubated with different concentrations of 7‐Epitaxol for 24 h. Afterwards, the cells were collected, fixed in 70% ice‐cold ethanol overnight and stained with PI buffer (4 mg/ml PI, 1% Triton X‐100, 0.5 mg/ml RNase A in PBS) for 30 min in the dark at room temperature. The cell cycle distribution was analysed by BD Accuri C6 Plus flow cytometry (BD Biosciences, San Jose, CA), and the data were analysed using BD CSampler Plus software.

### 2.6 DAPI staining

The cells were cultured in an 8‐well glass chamber slide at a density of 1 × 10^4^ cells/well overnight, followed by treatment with different concentrations of 7‐Epitaxol for 24 h. Afterwards, the cells were collected, fixed in 4% formaldehyde for 30 min and stained with DAPI dye (50 ug/ml) for 15 min in the dark. The nuclear morphological changes were assessed in at least 500 cells and photographed using Olympus FluoView FV1200 Confocal Microscope (Olympus Corporation, Shinjuku, Tokyo).

### 2.7 Annexin V/PI double staining assay

As previously described,^21^ the cell lines were treated with different concentrations of 7‐Epitaxol for 24 h. Then, the cells were harvested and suspended in PBS (2% BSA) and incubated with Muse Annexin V & Dead Cell reagent (EMD Millipore, Billerica, MA) for 20 min at room temperature in dark. The data were analysed by Muse Cell Analyzed flow cytometry (Merck Millipore, Burlington, MA).

### 2.8 Mitochondrial membrane potential measurement

As previously described,^21^ the cells were incubated with different concentrations of 7‐Epitaxol for 24 h. The cells were then collected and stained with Muse MitoPotential working solution at 37°C for 20 min. After incubating the cells with 5 μl of 7‐AAD for 5 min, Muse cell analyzer flow cytometer (EMD Millipore) was used to analyse the samples. The data were analysed by Muse Cell Analyzer (Millipore).

### 2.9 Western blot analysis

The cells were first treated with different concentrations of 7‐Epitaxol for 24 h, followed by lysis with RIPA buffer containing protease/phosphatase inhibitor cocktails to obtain cellular proteins. After measuring protein concentrations using BCA (Thermo Fisher Scientific) assay, the samples were separated using SDS‐PAGE and transferred to PVDF membranes (Millipore, Bedford, MA). The membranes were then blocked with 5% nonfat milk in TBST for 1 h, followed by incubation with appropriate primary and secondary antibodies (dilution ratio 1:1000) overnight at 4°C. The protein bands were visualized using enhanced chemiluminescence with an HRP substrate (Millipore).

### 2.10 In vivo antitumor growth effects of 7‐Epitaxol on xenograft transplantation

As previously described.^23^ For the experimental study of xenograft growth inhibition, 5–6‐week‐old male C57BL/6 mice (18–22 g) (National Taiwan University Animal Center, Taiwan) were used. Cis‐SAS cells (2 × 10^6^ per mouse) were resuspended in 100 μl of sterile PBS and injected sc into the right flank of the mouse. Mice were randomized into two groups (4 mice/group). All animals were housed with a regular 12‐h light/12‐h dark cycle and ad libitum access to standard rodent food diet (LaboratoryRodent Diet 5001, LabDiet, St. Louis, MO), and kept in a pathogen‐free environment in the Laboratory Animal Unit (temperature 22°C, humidity 30 ~ 70%, 5 mice/cage). 7 days after tumour cell injection, mice were fed 7‐Epitaxol (0.2 mg/kg) with 1 uM cisplatin three times a week. The control group received an equal volume of 0.5% carboxymethylcellulose vehicle with 1 uM cisplatin. Tumour volumes were determined from calliper measurements obtained every 3 days. At the end of the experiment, the mice were sacrificed and the primary tumours were removed for further analysis. Primary tumours were separated from the surrounding muscles and dermis and then weighed. Tumour volume was calculated using the following formula: 0.5 × length × width.^2^ The mean weight of the mice at the start of the study and at the end of the study did not differ significantly between the groups. All animal procedures were conducted according to the institutional animal welfare guidelines of the Institutional Animal Care and Use Committee (IACUC) of Changhua Christian Hospital.

### 2.11 Statistical analysis

The experimental data are expressed as means ± standard deviation. Each experiment was replicated at least three times. The statistical analyses were conducted by anova, Tukey's post hoc test and Student's *t*‐test. In all cases, a *p* value of < 0.05 was considered statistically significant. All statistical analyses were performed using Sigma‐Stat 2.0 (Jandel Scientific, San Rafael, CA).

## 3 RESULTS

### 3.1 Cytotoxic effects of 7‐Epitaxol in cisplatin‐resistant HNSCC

**FIGURE 1:**
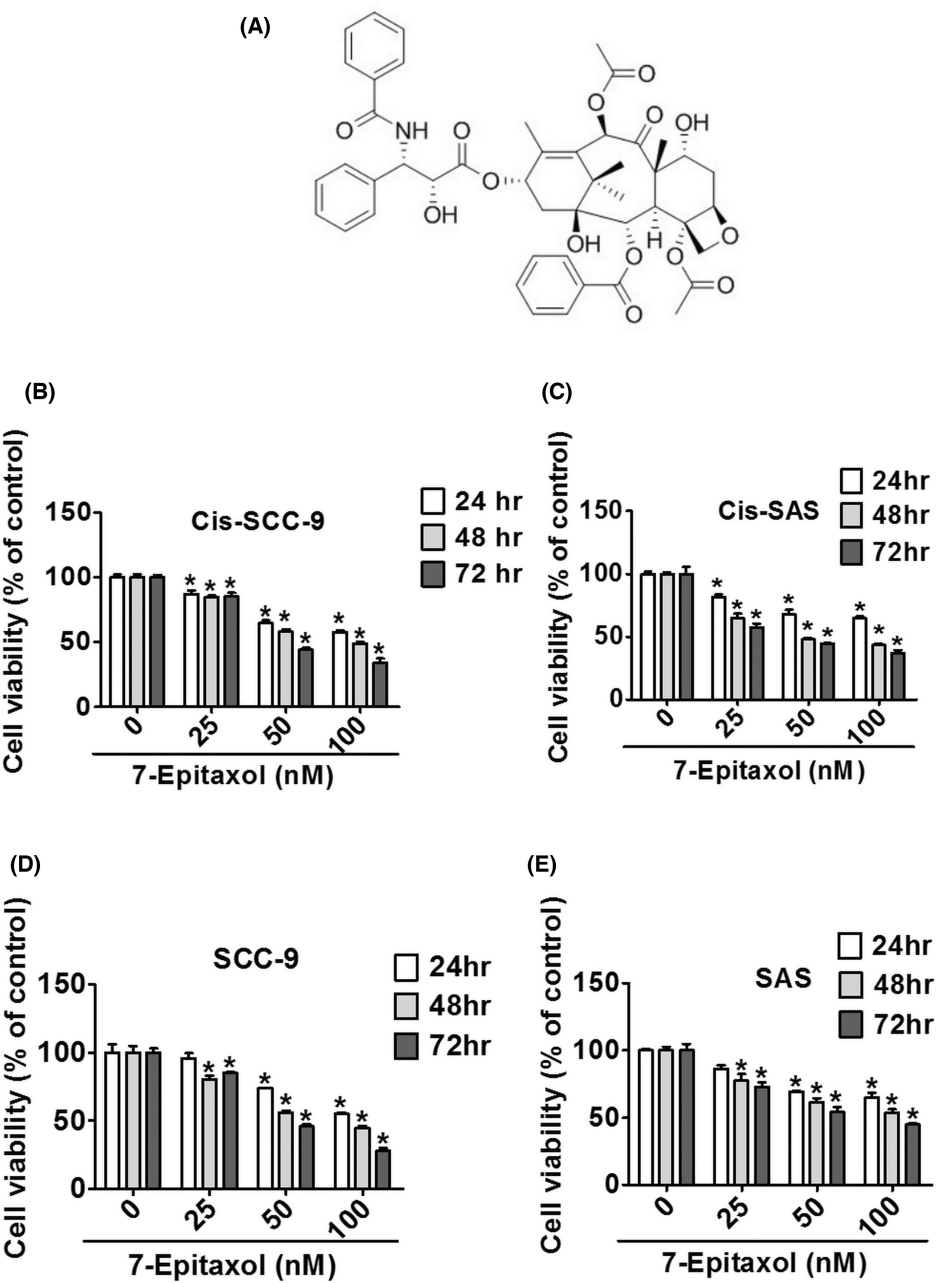
Cytotoxicity effects of 7‐Epitaxol in difference HNSCC cell lines. (A) The chemical structure of 7‐Epitaxol (7‐E). Cell viability was measured by MTT assay (B) Cis‐SCC‐9, (C) Cis‐SAS, (D) SCC‐9 and (E) SAS cells were treated with the indicated concentration of 7‐E (0, 25, 50 and 100 nM) for 24, 48 and 72 h. Data are presented as mean ± SD (*n* = 3). *p < 0.05, compared with the control group.

Cytotoxicity of 7‐Epitaxol (Figure 1A) was studied in two cisplatin‐resistant HNSCC cell lines SCC‐9 and SAS. The cells were treated with different concentrations of 7‐Epitaxol (0, 25, 50 and 100 nM) for 24, 48 and 72 h and subjected to MTT assay. The same HNSCC cell lines without cisplatin resistance were used as positive control. As observed in Figure 1B, C, the treatment with 7‐Epitaxol caused a dose‐dependent reduction in cell viability in both cisplatin‐resistant cell lines compared to that in untreated control cells. Similar effects were also observed in cells without resistance (Figure 1D, E). These findings highlight the cytotoxic effects of 7‐Epitaxol in both cisplatin‐resistant and non‐resistant HNSCC cells.

### 3.2 Effects of 7‐Epitaxol on cell cycle regulation in HNSCC

**FIGURE 2:**
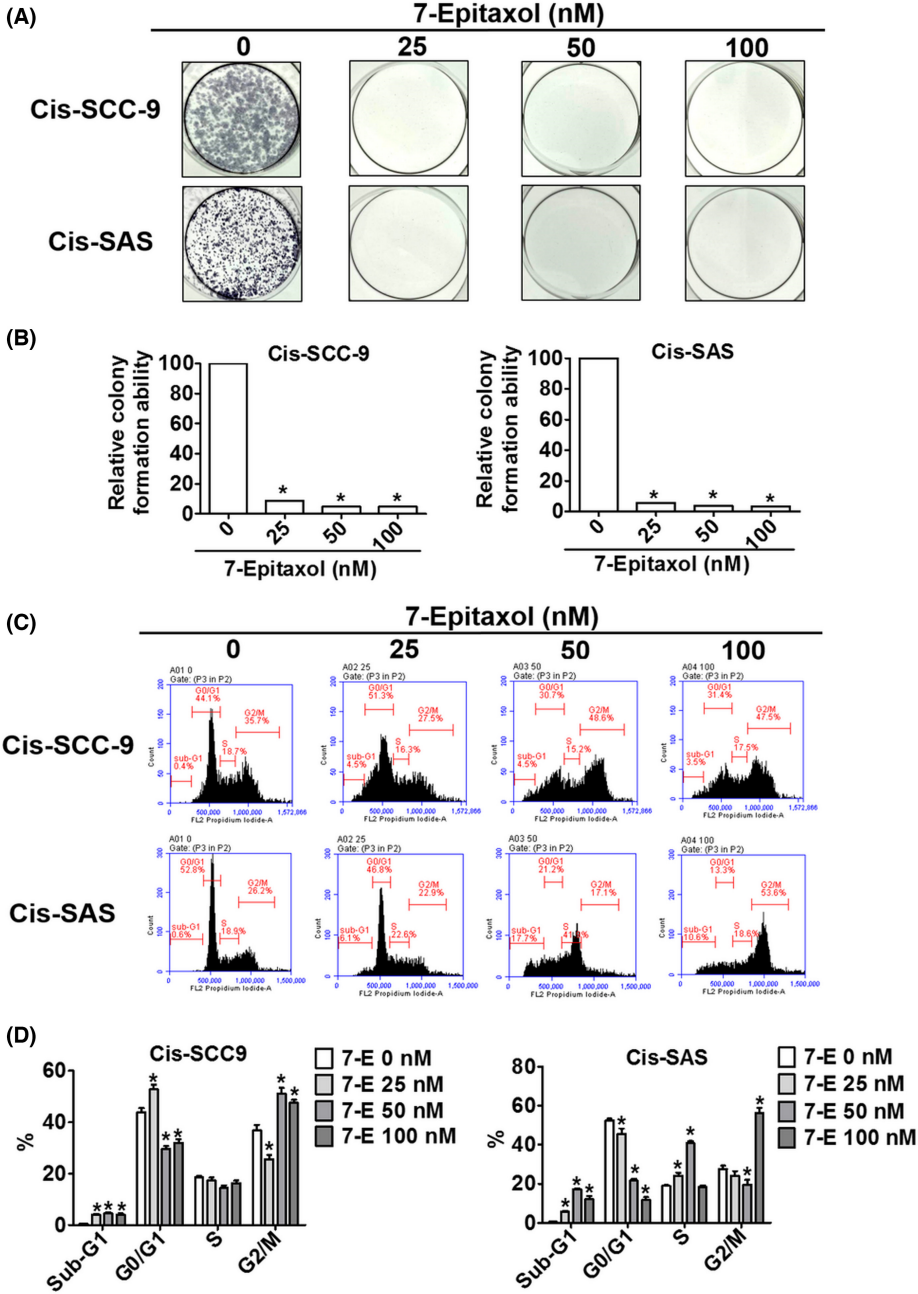
**7‐**Epitaxol induces cell cycle arrest and inhibits cell proliferation in Cis‐SCC9 and Cis‐SAS cells. (A, B) Cis‐SCC‐9 and Cis‐SAS were analysed by colony formation assay, that cells cultured in condition medium presence of 7‐E (0–100 nM) for 14 days. (C) After treated 7‐E (0–100 nM) for 24 h. Cells were used PI stained and flow cytometry performed to estimate cell cycle phase distribution. (D) Quantification of different cell cycle phase of sub‐G1, G0/G1, S and G2/M. Data are presented as mean ± SD (*n* = 3). *p < 0.05, compared with the control group.

Given the significant cytotoxic effects of 7‐Epitaxol, we next investigated its impact on cell cycle regulation and proliferation. Firstly, we performed colony formation assay using 7‐Epitaxol‐treated cisplatin‐resistant HNSCC cells to assess its ability to inhibit cancer cell proliferation. Afterwards, we performed flow cytometric analysis to cell cycle distribution of 7‐Epitaxol‐treated cells. As observed in Figure 2A, B, 7‐Epitaxol at all concentrations significantly reduced the colony formation ability of cancer cells. This finding further highlights the anti‐proliferative effect of 7‐Epitaxol in cisplatin‐resistant HNSCC cells. Regarding cell cycle regulation, 7‐Epitaxol was found to cause cell cycle arrest at the G2/M phase and reduce cell cycle rate at the G0/G1 phase in high concentration (50 and 100 nM in Cis‐SCC9 and 100 nM in Cis‐SAS). In addition, 7‐Epitaxol induces cell cycle rate at the sub‐G1 phase in both cisplatin‐resistant cell lines (Figure 2C, D). However, a variation in response to 7‐Epitaxol was observed between two cell lines at the S phase (Figure 2C, D). Overall, the findings indicate that 7‐Epitaxol reduces HNSCC proliferation by inducing cell cycle arrest.

### 3.3 Pro‐apoptotic effects of 7‐Epitaxol in HNSCC

**FIGURE 3:**
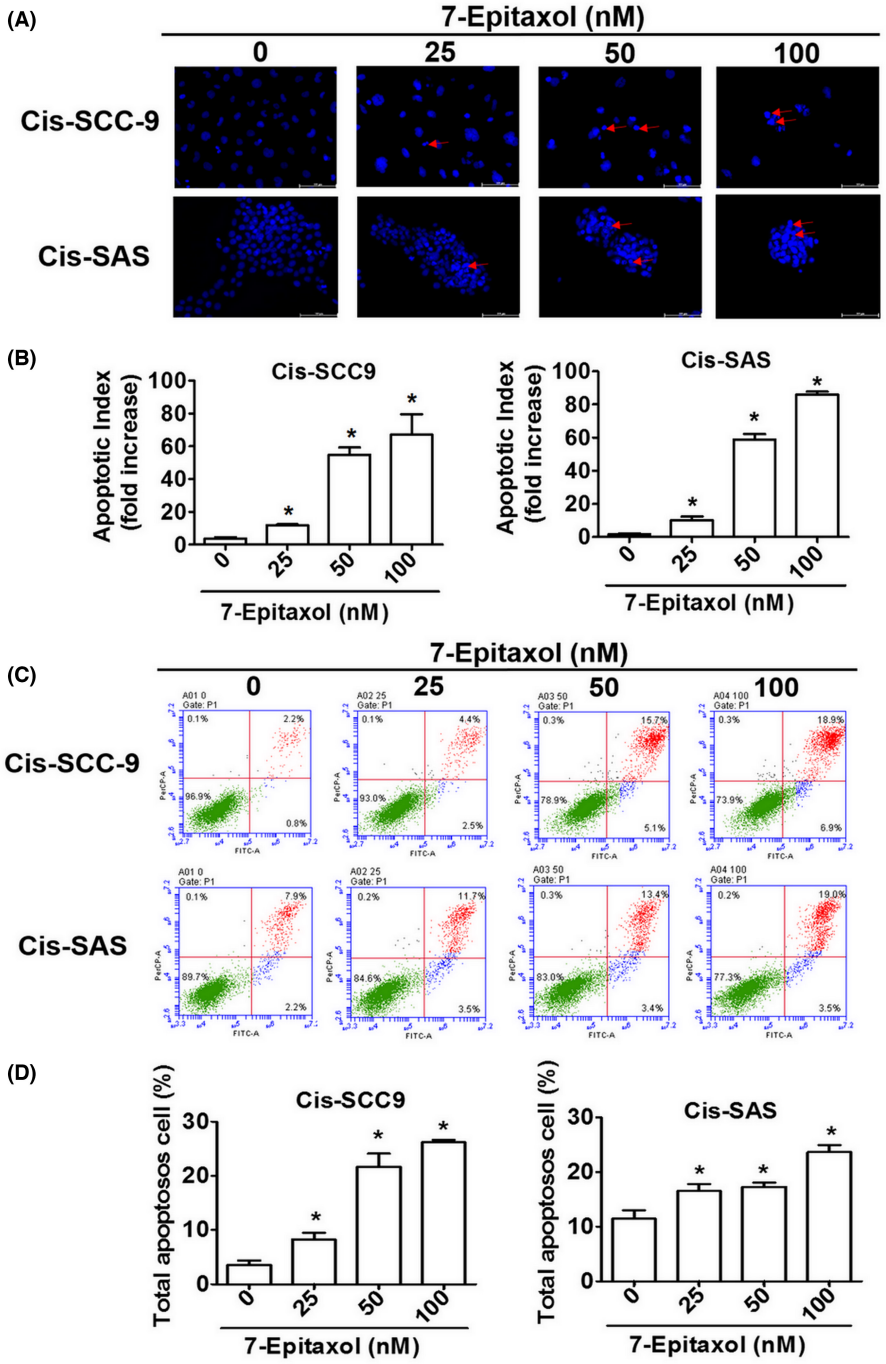
**7‐**Epitaxol induces apoptosis in Cis‐SCC9 and Cis‐SAS cells. After treated 7‐E (0–100 nM) for 24 h. (A, B) Used DAPI stain assay to determine DNA condensation with fluorescence microscopy. (C) Cell were stained Annexin V/PI and used flow cytometry to revealed 7‐E induced apoptosis. (D) Quantitative relative the percentage of apoptosis cells (including early and late state). Data are presented as mean ± SD (*n* = 3). *p < 0.05, compared with the control group.

Given the significant effect of 7‐Epitaxol on cell cycle regulation, we hypothesized that the compound might induce cell death in cisplatin‐resistant HNSCC cells. To prove our hypothesis, firstly we stained 7‐Epitaxol‐treated cells with DAPI and performed fluorescence microscopic analysis to evaluate morphological alterations and nuclear condensation. For further validation, we determined the percentage of apoptotic cells by staining the treated cells with Annexin V/PI staining and subsequently performing flow cytometry. As observed in Figure 3A, B, the 7‐Epitaxol treatment caused a significant increase in apoptotic index (ratio of total number of apoptotic bodies to total number of intact cancer cells) in a dose‐dependent manner in both cell lines compared to untreated cells. Moreover, a dose‐dependent increase in the percentage of apoptotic cells was observed following 7‐Epitaxol treatment (Figure 3C, D). Overall, these findings clearly highlight the pro‐apoptotic effects of 7‐Epitaxol in cisplatin‐resistant HNSCC.

### 3.4 Effects of 7‐Epitaxol on specific apoptotic pathways in HNSCC

**FIGURE 4:**
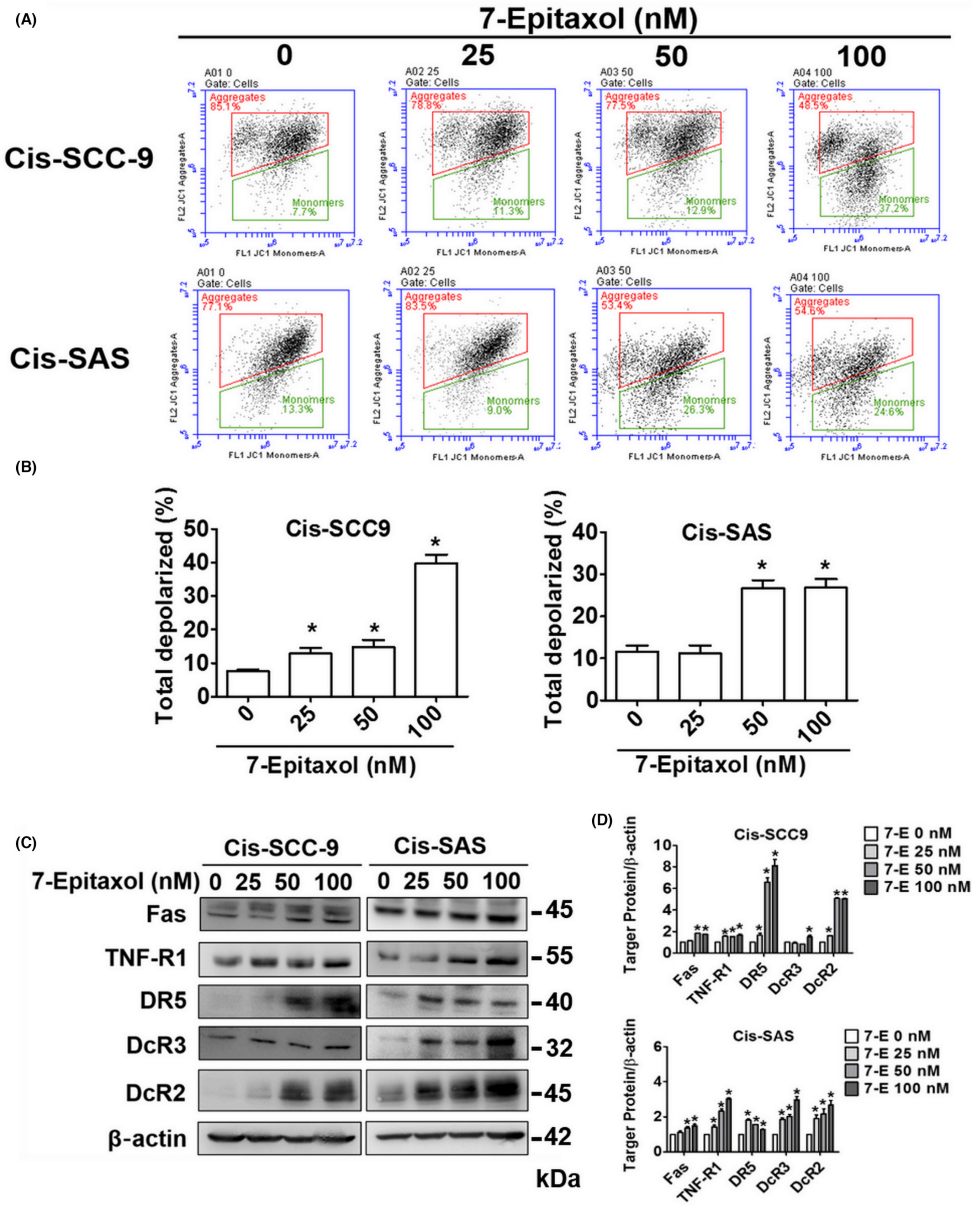
Intrinsic pathway and the extrinsic pathway were regulated by 7‐Epitaxol in Cis‐SCC9 and Cis‐SAS cells. After treated 7‐E (0–100 nM) for 24 h. (A) Mitochondrial membrane potential measurement assay was used by flow cytometry. (B) The data was analysed by Muse Cell Analyzer (Millipore). (C) Analyse the expression of extrinsic pathway control proteins, including Fas, TNF‐R1, DR5, DcR3, DcR2 and beta‐actin by Western blot. (D) Quantitative relative density of each protein levels was normalized to beta‐actin. Data are presented as mean ± SD (*n* = 3). *p < 0.05, compared with the control group.

**FIGURE 5:**
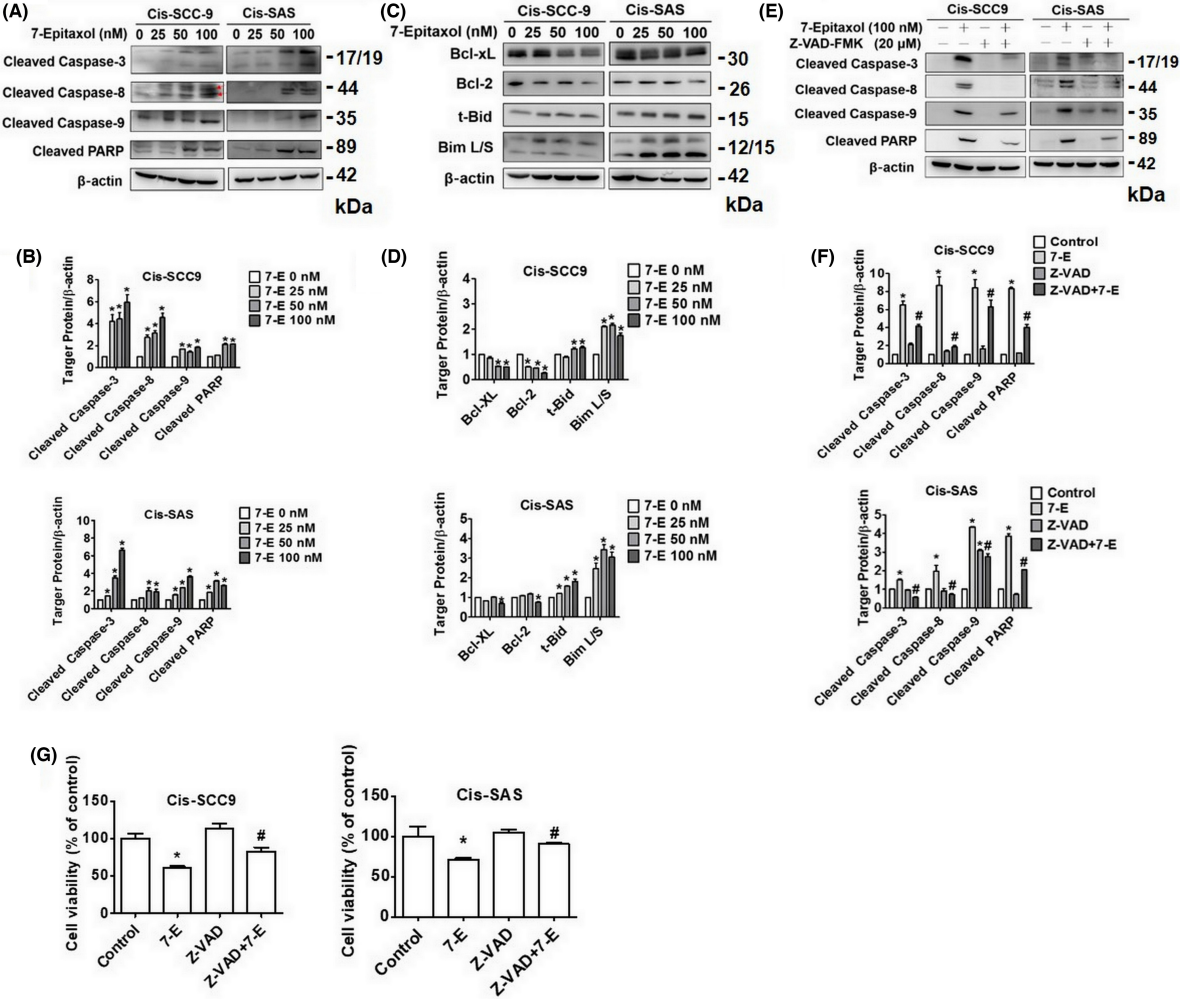
**7‐**Epitaxol activates caspase pathway and regulated Bcl‐2 family in Cis‐SCC9 and Cis‐SAS cells. Western blotting was used to measure the expression of regulated proteins after 24 h of 7‐E treatment in (A, B) the caspase pathway related proteins and (C, D) the Bcl‐2 family related proteins. (E, F) Western blotting was used to measure the expression of caspase pathway related proteins after 24 h of Z‐VAD‐FMK (Z‐VAD) (20 μM) combined with 7‐E in Cis‐SCC9 and Cis‐SAS cells. (G) Cell viability was measured by MTT assay. Quantitative relative density of each protein levels was normalized to beta‐actin. Data are presented as mean ± SD (*n* = 3). *p < 0.05, compared with the control group.

Considering the apoptotic‐inducing ability of 7‐Epitaxol, we next thought of identifying specific apoptotic pathways involved. Given the significant involvement of mitochondria in mediating apoptosis, we firstly determined mitochondrial membrane potential in 7‐Epitaxol‐treated cells. Afterwards, we performed Western blot analyses to determine whether 7‐Epitaxol can alter the expressions of proteins related to both intrinsic and extrinsic apoptotic pathways. As observed in Figure 4A, B, 7‐Epitaxol treatment significantly increased mitochondrial membrane depolarization in both cell lines in a dose‐dependent manner. Moreover, compared to untreated cells, 7‐Epitaxol‐treated cells showed significantly higher expressions of extrinsic pathway proteins including FAS, TNF‐R1, and death and decoy receptors (DR5, DcR3 and DcR2). Regarding intrinsic pathway, 7‐Epitaxol significantly increased the expressions of pro‐apoptotic proteins Bid and Bim L/S and decreased the expressions of anti‐apoptotic proteins Bcl‐2 and Bcl‐XL (Figure 5C, D). Since both intrinsic and extrinsic apoptotic pathways are mediated by activation and cleavage of caspases, we also determined the expressions of PARP and caspases 3, 8 and 9 in 7‐Epitaxol‐treated cells. The findings revealed that 7‐Epitaxol treatment significantly increased the expressions of all tested caspases and PARP (Figure 5A, B). To further confirm the mechanism of apoptosis of 7‐Epitaxol in cisplatin‐resistant HNSCC cells, Cis‐SCC‐9 and Cis ‐SAS cells were treated with Z‐VAD‐FMK (pan caspase inhibitor) and 7‐Epitaxol. The increased expression of cleaved caspases 3, 8 and 9 and PARP induced by 7‐Epitaxol were suppressed in the 7‐Epitaxol/Z‐VAD‐FMK combined group (Figure 5E, F). Furthermore, inhibition of apoptosis almost completely reverses 7‐Epitaxol‐induced cell death (Figure 5G). Overall, these findings confirm that 7‐Epitaxol induces apoptosis in cisplatin‐resistant HNSCC by inducing intrinsic and extrinsic pathways and activating PARP and caspases 3, 8 and 9.

### 3.5 Effects of 7‐Epitaxol on AKT and MAPK signalling pathways in HNSCC

**FIGURE 6:**
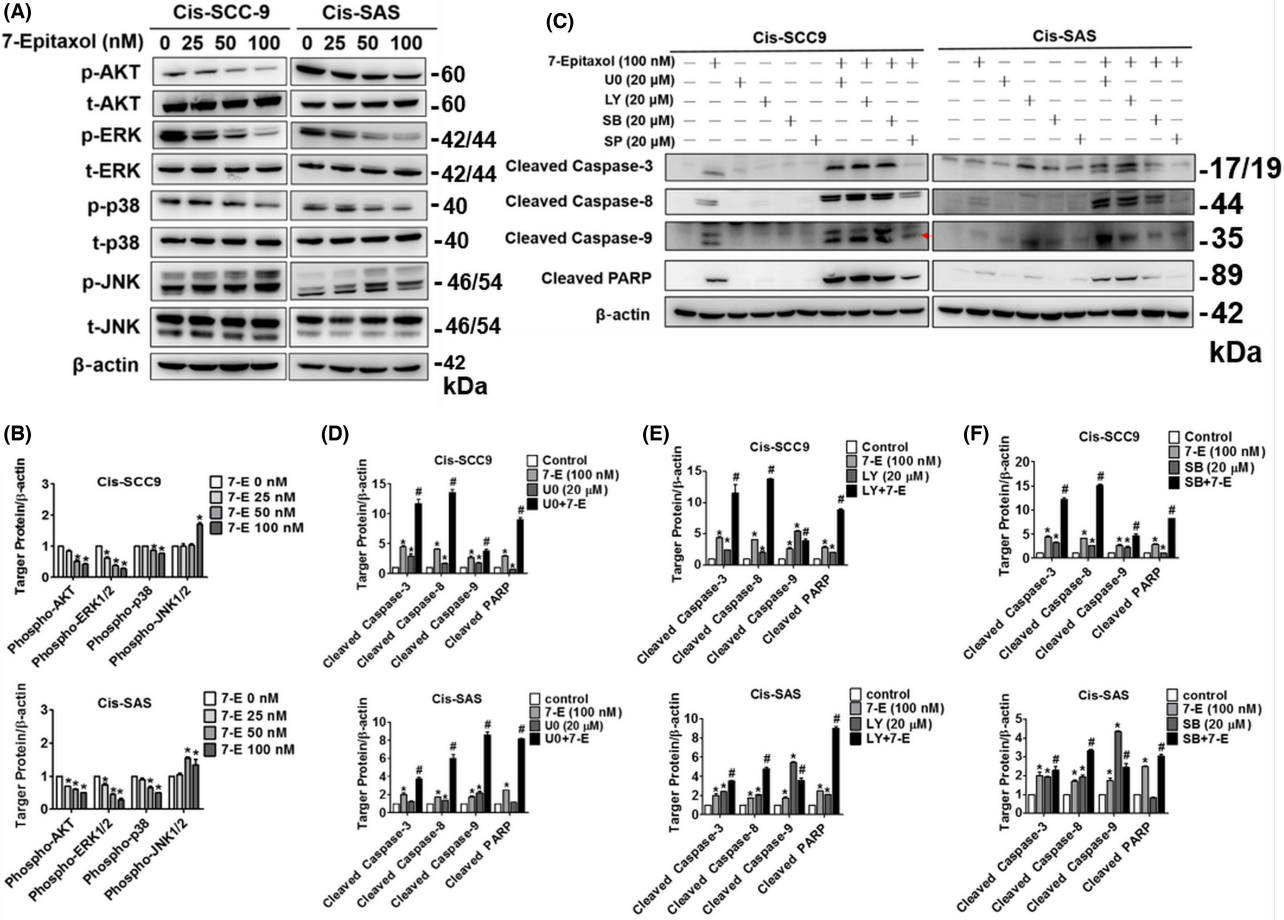
7‐Epitaxol induces apoptosis by affecting the AKT, ERK1/2 and p38 MAPK pathway in Cis‐SCC9 and Cis‐SAS cells. Cell lines were pre‐treatment with specific inhibitors (U0126, LY294002, SB203580, SP600125) or without for 1 h, then treated 7‐E for 24 h. Western blotting was used to measure the expression of regulated proteins (A, B) the AKT and MAPK pathway, (C‐F) the caspase pathway proteins. Quantitative relative density of each protein levels was normalized to beta‐actin. Data are presented as mean ± SD (*n* = 3). *p < 0.05, compared with the control group. #*p* < 0.05, compared with the cells treated with 7‐E (100 nM).

To identify the cellular signalling pathways involved in 7‐Epitaxol‐mediated apoptosis, we determined the expressions of AKT and MAPK pathway components (ERK1/2, p38 and JNK) in 7‐Epitaxol‐treated cells. As observed in Figure 6A, B, 7‐Epitaxol treatment significantly reduced the phosphorylation of AKT, ERK1/2 and p38 in both cell lines. In contrast, a significantly increased phosphorylation of JNK was observed when the cells were treated with higher concentration of 7‐Epitaxol (Figure 6A, B). For further validations, the cells were pretreated with specific inhibitors of ERK1/2 (U0126), AKT (LY294002), p38 (SB203580) and JNK (SP600125), followed by treatment with 7‐Epitaxol. In these co‐treated cells, we determined the expressions of PARP and caspases 3, 8 and 9. Interestingly, we observed that the cotreatment with AKT, ERK1/2 and p38 inhibitors further increased the expressions of cleaved PARP and caspases 3, 8 and 9, as compared to 7‐Epitaxol treatment alone (Figure 6C–F). These findings confirm that 7‐Epitaxol activates caspase‐mediated apoptotic signalling pathways by suppressing the activation of AKT, ERK1/2 and p38.

### 3.6 Significant anti‐proliferative effects of 7‐Epitaxol in the orthotopic graft model of HNSCC resistant to cisplatin

**FIGURE 7:**
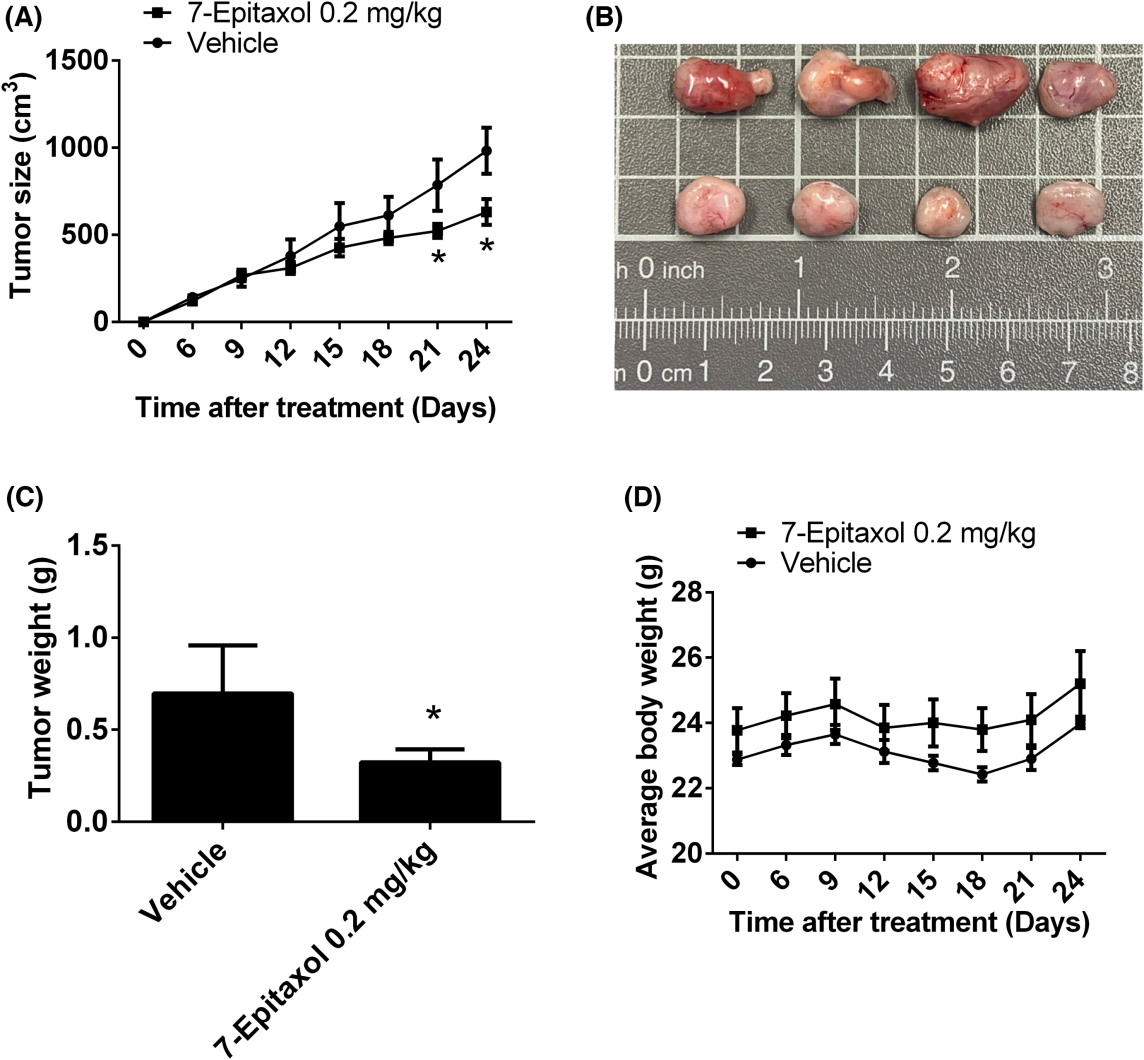
7‐Epitaxol suppressed tumour growth of Cis‐SAS cells in vivo. (A, B) Cis‐SAS cells were injected into the right flank of 6‐week‐old male nude mice. After injection of Cis‐SAS cells, nude mice were treated with vehicle or 7‐Epitaxol (0.2 mg/kg) with 1 uM cisplatin three times a week. The growth of the xenograft tumours was referred to the measurement of the long and short dimensions of the tumours, and the calculation of the tumour size was described in the section ‘Materials and Methods’. (C) Tumour weight and (D) body weight changes of the mice during treatment. *p < 0.05, compared to vehicle.

To test the effect of 7‐epitaxol on tumour growth, the in vivo antitumor effect of 7‐epitaxol was evaluated. Tumour volumes were determined by calliper measurements every 3 days. The control group of animals that transplanted Cis‐SAS cancer cells showed a progressive increase in their tumour volumes. In mice treated with 7‐epitaxol receiving 0.2 mg/kg with cisplatin, the mean tumour volume (Figure 7A, B) and tumour weight (Figure 7C) were significantly inhibited compared to vehicles treated. As illustrated in Figure 7D, no significant differences in body weight were detected among these groups. These results showed that animals treated with 7‐Epitaxol show antitumor growth compared to control animal*s*.

## 4 DISCUSSION

The present study has been designed to investigate the anticancer effects and mode of action of 7‐Epitaxol, a major metabolite of paclitaxel with higher stability and cytotoxicity, in cisplatin‐resistant HNSCC. There are many studies on the effect of paclitaxel, but research on 7‐epitaxol in HNSCC is still limited. Paclitaxel is an agent that stabilizes microtubules and prevents cell mitosis in the G2/M phase. It is widely used to treat many cancers including breast cancer, ovarian cancer, endometrial cancer, lung cancer, bladder cancer and cervical cancer.^24^ 7‐Epitaxol is an active hydrolytic compound of paclitaxel. The ester groups and a chiral centre of paclitaxel undergo epimerization at the 7 position and then form 7‐Epitaxol, which shows comparable properties for microtubule polymerization in vitro.^25^ Based on the study findings, 7‐Epitaxol exerts significant cytotoxicity and reduces the proliferation of HNSCC cells (Figure 1). The anti‐proliferative effect of 7‐Epitaxol is mediated through the induction of cell cycle arrest at G0/G1 and G2/M phase and activation of intrinsic and extrinsic apoptotic pathways (Figures 2, 3, 4, 5). Specifically, the study describes that 7‐Epitaxol induces the expression of pro‐apoptotic proteins and suppresses the expression of anti‐apoptotic proteins to trigger caspase‐mediated apoptotic signalling (Figures 4 and 5). Regarding upstream cellular signalling, the study finds that 7‐Epitaxol reduces the phosphorylation of AKT, ERK1/2 and p38 in cisplatin‐resistant HNSCC cells, leading to cleavage and activation of PARP and caspases 3, 8 and 9 and subsequent induction of apoptosis (Figure 6).

Our findings are in line with a previous study that shows 7‐Epitaxol inhibits cell viability of HNSCC cells through arresting the cell cycle and induce cell death, depolarization of mitochondria and loss of mitochondrial membrane potential.^21^ As observed in the study, 7‐Epitaxol modulates the expressions of intrinsic and extrinsic pathway proteins and induces the activation of PARP and caspases 3, 8 and 9 to trigger cancer cell apoptosis. Moreover, the study demonstrates that 7‐Epitaxol mediates its pro‐apoptotic effects by reducing the phosphorylation of AKT and ERK1/2. In the present study, we have observed similar findings in cisplatin‐resistant HNSCC cells, indicating that 7‐Epitaxol could exert anticancer effects in cancer cells irrespective of their status of resistance to other potent chemotherapeutic agents like cisplatin.

According to the available literature, paclitaxel induces cell cycle arrest at the G2/M phase by stabilizing microtubules and preventing their disassembly during cell division.^26^, ^27^ These findings justify the cancer cell growth‐arresting effects of 7‐Epitaxol we observed in the present study (Figure 2). Furthermore, paclitaxel has been found to induce cancer cell apoptosis by regulating the expression of pro‐ and anti‐apoptotic proteins, altering mitochondrial membrane potential, triggering intrinsic pathway components and subsequently activating caspase 3.^28^, ^29^, ^30^ These findings are also in line with our observation about the pro‐apoptotic mode of action of 7‐Epitaxol in cisplatin‐resistant HNSCC cells.

Aberrant activation of the AKT/PI3K and MAPK pathways is a major hallmark in many cancers.^31^, ^32^ Given the significance of these pathways in modulating cancer cell proliferation, survival, angiogenesis and metastasis,^33^, ^34^ we investigated whether 7‐Epitaxol treatment alters the expressions of AKT/PI3K and MAPK pathway components. We observed a significant reduction in AKT, ERK1/2 and p38 phosphorylation (Figure 6). Moreover, the treatment of cells with respective inhibitors of these components along with 7‐Epitaxol caused a further activation of caspases and PARP compared to that caused by 7‐Epitaxol treatment alone (Figure 6).

In line with our findings, a previous study has shown that 7‐Epitaxol induces apoptosis of HNSCC cells by reducing the phosphorylation of ERK1/2 and AKT.^21^ Interestingly, there is evidence that activation of p38 regulates apoptosis and results in cell death. However, our results show that 7‐Epitaxol inactivates p38 in cisplatin‐resistant HNSCC cells, leading to induction of apoptosis. Here, we investigate the role of p38 in the apoptosis of cisplatin‐resistant HNSCC cells treated with 7‐Epitaxol. In the early stage of tumorigenesis, activation of p38α induced apoptosis; however, in the advanced stage of tumour, apoptosis seems to be regulated by the JNK pathway, which sensitizes tumour cells to drug‐induced apoptosis. Pereira et al. illustrated that inhibition of p38 MAPK signalling sensitizes tumour cells to cisplatin‐induced apoptosis by up‐regulation of ROS and activation of the JNK pathway by inhibiting phosphatase activity.^35^ Activation of p38 MAPK has been reported in most HNSCC cells, and interruption of p38 signalling resulted in suppression of tumour cell proliferation and survival both in vitro and in vivo. It also suggested that, compared to cisplatin plus the p38 MAPK inhibitor versus cisplatin alone, the former exhibited a significant decrease in cell proliferation and enhanced apoptosis in HNSCC cells.^36^

Furthermore, inhibition of p38 in cisplatin‐resistant HNSCC cells reduced the expression of cancer stem cell makers, β‐catenin, migration and sphere formation ability along with increased apoptotic index, and sensitizes these resistant cells to cisplatin. In conclusion, the study suggested that inactivation of p38 MAPK could improve the efficacy of chemotherapy in drug‐resistant HNSCC.^37^ Our experimental result that 7‐Epitaxol can reduce p38 expression and further increase therapeutic efficacy in cis‐resistant HNSCC cells could be confirmed by these previous studies. One recent study has demonstrated that dual inhibition of AKT/PI3K and MAPK signalling increases the sensitivity of pancreatic ductal adenocarcinoma to paclitaxel/gemcitabine chemotherapy.^38^ Similarly, a phase II randomized trial has recently revealed that a combination therapy with paclitaxel and an MAPK/extracellular signal‐regulated kinase (MEK) inhibitor (cobimetinib) increases progression‐free survival in patients with metastatic triple‐negative breast cancer.^39^ These observations clearly indicate that the sensitivity of cancer cells to chemotherapeutic agents can be increased through the inhibition of AKT/PI3K and MAPK signalling. In our study, this could be a possible mechanism of high anticancer efficacy of 7‐Epitaxol against HNSCC cells that are otherwise resistant to another chemotherapeutic agent cisplatin.

## 5 CONCLUSION

The study demonstrates the anticancer efficacy of a major paclitaxel metabolite, 7‐Epitaxol, in cisplatin‐resistant HNSCC. The findings reveal that 7‐Epitaxol reduces cell viability by inducing cell cycle arrest and apoptosis in HNSCC cell lines. Mechanistically, 7‐Epitaxol causes mitochondrial membrane depolarization and activation of both intrinsic and extrinsic apoptotic pathways. As observed in the study, 7‐Epitaxol increases the expressions of pro‐apoptotic proteins Bid and Bim L/S and reduces the expressions of anti‐apoptotic proteins Bcl‐2 and Bcl‐XL, leading to activation of PARP and caspases 3, 8 and 9 and subsequent induction of cancer cell apoptosis. At the cell signalling level, 7‐Epitaxol reduces the phosphorylation of AKT, ERK1/2 and p38 to induce caspase‐mediated apoptosis. Taken together, the study identifies 7‐Epitaxol as a potent anticancer agent that might be used to treat cisplatin‐resistant HNSCC.

## AUTHOR CONTRIBUTIONS

**Hui‐Ju Yang:** Conceptualization (equal); writing – original draft (equal); writing – review and editing (equal). **Bharath Kumar Velmurugan:** Conceptualization (equal); writing – original draft (equal). **Mu‐Kuan Chen:** Conceptualization (equal); resources (equal). **Chia‐Chieh Lin:** Methodology (equal). **Yu‐Sheng Lo:** Methodology (equal). **Yi‐Ching Chuang:** Methodology (equal). **Hsin‐Yu Ho:** Methodology (equal). **Ming‐Ju Hsieh:** Conceptualization (equal); writing – review and editing (equal). **Jiunn‐Liang Ko:** Conceptualization (equal); writing – review and editing (equal).

## FUNDING INFORMATION

This research received no external funding.

## CONFLICT OF INTEREST

The authors declare no conflicts of interest.

## Data Availability

Data availability statementThe data that support the findings of this study are available from the corresponding author upon reasonable request.
